# miR-29b and miR-198 overexpression in CD8^+^ T cells of renal cell carcinoma patients down-modulates JAK3 and MCL-1 leading to immune dysfunction

**DOI:** 10.1186/s12967-016-0841-9

**Published:** 2016-04-11

**Authors:** Margherita Gigante, Paola Pontrelli, Wolfgang Herr, Maddalena Gigante, Morena D’Avenia, Gianluigi Zaza, Elisabetta Cavalcanti, Matteo Accetturo, Giuseppe Lucarelli, Giuseppe Carrieri, Michele Battaglia, Walter J. Storkus, Loreto Gesualdo, Elena Ranieri

**Affiliations:** Department of Emergency and Organ Transplantation, University of Bari, Bari, Italy; Department of Medicine III, Hematology and Oncology, University Medical Center of Regensburg, Regensburg, Germany; Department of Medical and Surgical Sciences, Faculty of Medicine, University of Foggia, Viale Luigi Pinto, 1, 71100 Foggia, Italy; Department of Internal Medicine and Oncology, University of Bari, Bari, Italy; Renal Unit, Department of Medicine, University of Verona, Verona, Italy; Department of Dermatology and Immunology, University of Pittsburgh, Pittsburgh, PA USA

**Keywords:** Renal cell carcinoma, CD8^+^ T cells, Apoptosis, miRNA, JAK3, MCL-1

## Abstract

**Background:**

Mammalian microRNAs (miR) regulate the expression of genes relevant for the development of adaptive and innate immunity against cancer. Since T cell dysfunction has previously been reported in patients with renal cell carcinoma (RCC; clear cell type), we aimed to analyze these immune cells for genetic and protein differences when compared to normal donor T cells freshly after isolation and 35 days after in vitro stimulation (IVS) with HLA-matched RCC tumor cells.

**Methods:**

We investigated gene expression profiles of tumor-reactive CD8^+^ T cells obtained from RCC patient and compared with their HLA-matched healthy sibling donors using a microarray approach. In addition, miRNAs analysis was performed in a validation cohort of peripheral blood CD8^+^ T cells from 25 RCC patients compared to 15 healthy volunteers.

**Results:**

We observed that CD8^+^ T cells from RCC patients expressed reduced levels of anti-apoptotic and proliferation-associated gene products when compared with normal donor T cells both pre- and post-IVS. In particular, JAK3 and MCL-1 were down-regulated in patient CD8^+^ T cells versus their normal counterparts, likely due to defective suppressor activity of miR-29b and miR-198 in RCC CD8^+^ T cells. Indeed, specific inhibition of miR-29b or miR-198 in peripheral blood mononuclear cells (PBMCs) isolated from RCC patients, resulted in the up-regulation of JAK3 and MCL-1 proteins and significant improvement of cell survival in vitro.

**Conclusions:**

Our results suggest that miR-29b and miR-198 dysregulation in RCC patient CD8^+^ T cells is associated with dysfunctional immunity and foreshadow the development of miR-targeted therapeutics to correct such T cell defects in vivo.

**Electronic supplementary material:**

The online version of this article (doi:10.1186/s12967-016-0841-9) contains supplementary material, which is available to authorized users.

## Background

MicroRNAs (miRs) are 19- to 25-nucleotide non-coding RNA molecules that regulate gene expression at the level of transcription and translation by sequence-specific base pairing on the 3*′* UTR of mRNA targets [1]. Findings over the past several years have revealed the important role of miRNAs in the regulation of crucial biological processes, including cell growth, differentiation, proliferation and apoptosis [2, 3]. More recently, numerous studies have demonstrated that miRNA profiles dictate the nature of adaptive and innate immunity, including controlling the differentiation of various immune cell subsets and their biologic functions [4, 5]. Aberrant expression of miRNAs has also been reported in the context of different types of human cancers [6] such as lung cancer [7], breast cancer [8], gastric cancer [9], and also renal cell carcinoma (RCC) [10, 11].

Clear-cell RCC is the most common type of kidney cancer, whose incidence rate has notably increased in recent years. RCC is perceived as an immunogenic tumor, in that spontaneous regression and/or objective clinical responses to immunotherapy have been observed in a minority of cases [12–14]. However, in the vast majority of RCC patients, T cell responses are believed to be dysfunctional or “functionally inappropriate”, with many T effector cells noted to be in a pro-apoptotic state [15, 16]. We reported that CD8^+^ T cells isolated from RCC patients exhibit defects in the JAK3/STAT5/6-signaling pathway, leading to T cell arrest in the G0/G1 phase of the cell cycle and prevention of their terminal differentiation [17]. In the current study, we determined that *JAK3* and *MCL*-*1* expression is dysregulated in patient versus normal donor CD8^+^ T cells. MCL-1 is well known to be an anti-apoptotic member of the Bcl-2 family that promotes cell viability proliferation and differentiation [18, 19]. JAK3 is a non-receptor tyrosine kinase that has been implicated in the signal transduction of the common gamma chain subfamily of cytokine receptors. As a result, JAK3 plays an essential role in development, differentiation, proliferation and survival of T-cells [20] and, as we have previously reported, JAK3 mutations are associated with metastatic spread and poor survival of RCC patients [21].

Notably, we determined that MCL-1 and JAK3 expression levels in T cells were counter-regulated by miR-29b and miR-198, respectively, in RCC patients. These disease-associated miR alterations may hamper the effectiveness of anti-tumor T cell responses and serve as biomarkers for T cell dysfunction in the cancer setting. Our data support the development of specific miR-targeted therapeutics to resurrect/salvage anti-tumor T cell function in patients with RCC.

## Methods

### Patient samples

Peripheral blood mononuclear cells (PBMC) and well-differentiated RCC of the clear cell type (showing prominent cytoplasmic clearing and thin-walled vascular channels) were obtained with written consent from 25 patients treated with open radical nephrectomy or nephron-sparing surgery for clinically localized sporadic disease. Tumor cell cultures were established as previously described [22] using RPMI 1640 media supplemented with 2 mM l-glutamine 100 IU/ml penicillin and 100 μg/ml streptomycin (Life Technologies, Inc. Grand Island, NY) and 20 % fetal bovine serum (FBS, Sigma). Based on its preferred growth characteristics, patient TC was selected and genotyped as HLA-A3, -A24, -B7, -B8 and Cw 7 (homozygous). The cloned RCC cell line developed from patient TC has been patented TC tumor-derived cell line (ELTHEM —International patent PCT/EP2006-067631: renal cell carcinoma cell line and use thereof), and represents a highly-immunogenic cell line.

### PBMCs and CD8^+^ T cells isolation

PBMCs were isolated at the interface of Ficoll-Hypaque density gradients after centrifugation (Sigma Chemical Co., St Louis, MO) from RCC patients and HLA-matched healthy donors, following the manufacturer’s instructions. Cells were washed twice in PBS 1X (Invitrogen-Life Technologies, Italy) and used in mixed lymphocyte/tumor cell cultures as described below. HLA Class I typing was done using sequence-specific primed PCR (PCR-SSP) from genomic DNA (Dr. B. Favoino, Tissue Typing Laboratory, Department of Clinical Pathology II, Bari, Italy). HLA-matched normal donors were selected based on their sharing of single MHC class I alleles with the TC RCC cell line (Table 1). In particular, healthy donor 2 was a sibling donor. CD8^+^ T cells were isolated from the PBMCs of autologous and allogeneic normal, healthy donors by negative selection using specific MACS magnetic beads (Miltenyi Biotec, Bergisch Gladbach, Germany) on MiniMACS columns, following the manufacturer’s protocol and subsequently assessed for their functional and phenotypic profiles.

**Table 1:** HLA-class I genotyping of TC RCC and healthy donor-2 and donor-3 PBMCs included in MLTC

	HLA–A^*^	HLA–B^*^	HLA–C^*^
TC RCC	03:01 24:02	07:02 08:01	07:02 07:02
Donor-2	03:01 24:02	07:02 08:01	07:02 07:02
Donor-3	03:01 24:02	07:02 08:01	07:02 07:02

### Mixed lymphocyte/tumor cell cultures (MLTC)

MLTC experiments were performed as described in detail elsewhere (17,23). Briefly, PBMCs from RCC patients or HLA-matched healthy donors were co-cultured in 24-well plates (Costar, Corning, USA) at 10^6^ cells/well with irradiated RCC stimulator cells (10^5^ cells/well) in AIM-V medium (Life Technologies, Invitrogen, Italy) supplemented with 10 % heat-inactivated pooled human serum [Sigma (medium Mb)]. Recombinant human IL-2 was added on day 3 (250 IU/mL; Proleukin, Chiron, and Emeryville, CA). Responder lymphocytes were restimulated weekly with 10^5^ irradiated tumor cells in IL-2-containing medium for 2 weeks prior to responder CD8^+^ T cells isolation. Positively isolated CD8^+^ T cells were cultured for an additional 2 weeks. On day 0 and day 35 of culture, CD8^+^ T cells were analysed for gene and miRNA expression profiles.

### Flow cytometry

One million freshly-isolated or day 35 cultured CD8^+^ T cells were washed twice with cold PBS 1× and double-stained with Annexin V/7-AAD (Beckman Coulter). The cells were then analysed by a Beckman Coulter (FC500) flow cytometer. The same experiment was performed on PBMC at 24 and 48 h after transfection with anti-hsa-miR-29b and anti-hsa-miR-198 and stained with Annexin V/propidium iodide (PI) (Beckman Coulter). Apoptotic cells were considered as the sum of early and late apoptotic cells; early apoptotic cells are Annexin-V^+^/7-AAD^–^ or IP^−^; late apoptotic cells are both Annexin-V^+^7/7-AAD^+^ or IP^+^; and necrotic cells were Annexin-V^−^/7-AAD^+^ or IP^+^. Jurkat T cells treated with dexamethasone 10^−4^ M for 24 h at 37 °C served as a positive control.

### Microarray analysis and gene expression profiling

Total RNA was isolated from freshly-isolated and day 35 cultured CD8^+^ T cells using Trizol© reagent (Life Technologies), according to the manufacturer’s instructions. Total RNA integrity was assessed by electrophoresis using the Agilent 2100 Bioanalyzer (Agilent, Palo Alto, CA). Total RNA was processed and hybridized on to the GeneChip Human Genome U133A Array (Affymetrix, Santa Clara, CA), which contains 22,283 gene probe sets, representing approximately 12,000 well-characterized human genes (Affymetrix, Santa Clara, CA). For the statistical analysis, gene expression values for all gene probe sets, scaled to the target intensity of 2500, were log2 transformed. Then, the analysis was carried out using the weighted average (WPGMA) method. Principal component analysis (PCA) and 2-D hierarchical clustering were performed using Spotfire DecisionSite 8.1.1 (www.spotfire.com). Differentially expressed genes were identified using GeneSpring GX software (Agilent, Palo Alto, CA), by applying a moderated *t* test with Storey with bootstrapping multiple testing correction. The fold change (FC) were obtained as ratio of normalized intensities between the average intensities of the samples grouped. To assess the relationship between genes, Ingenuity Pathways Analysis (IPA) (Ingenuity^®^ Systems, www.ingenuity.com), a software which constructs content rich relationship networks between genes based on their connectivity according to information contained in the ingenuity pathways knowledge base, was applied. Results of the microarray experiments are available in Gene Expression Omnibus (Accession numbers: GSE6357).

### Western blot validation of gene expression

CD8^+^ T cells were lysed with RIPA buffer (1 mM phenylmethylsulphonylfluoride [PMSF], 5 mM EDTA, 1 mM sodium orthovanadate, 150 mM sodium chloride, 8 μg/ml leupeptin, 1.5 % Nonidet P-40, 20 mM tris–HCl, pH 7.4) and 40 μg of each lysate were separated by SDS-PAGE and electroblotted onto a nitrocellulose filter. The filter was blocked overnight at RT with 10 % BSA in PBS containing 0.1 % Tween-20 (TBS) and incubated with anti-human JAK3 and anti-human MCL-1 Abs in 5 % BSA-TBS (clones B-12 and B-6 respectively; 1:250 dilution, Santa Cruz Biotechnology) overnight, at 4 °C. The membranes were washed in TBS-T 0.1 % and incubated for 1 h at RT with horseradish-peroxidase-conjugated sheep anti-mouse IgG at 1:5000 dilution in TBS-T 0.1 %. The membrane was then visualized using the ECL enhanced chemiluminescence system (Amersham, Little Chalfont, UK). The same membranes were then immunoblotted again with anti-actin antibody (clone MM2/193; 1:2000 dilution; Santa Cruz Biotechnology). Signal detection and densitometric analysis of the bands was performed using a UVIchemi (UVItec, Cambridge) digital imaging system and UVI1D software (UVItec, Cambridge).

### Reverse transcription and microRNA Real-Time PCR quantification

CDNA was synthesized from total RNA of RCC CD8^+^ T cells using gene-specific primers according to theTaqMan MicroRNA assay protocol (PE Applied Biosystems, Foster City, CA). Reverse reactions for miR-29b and miR-198 were performed using 10 ng of RNA samples, 50 nM stem-loop RT primers, 1× RT buffer, 0.25 mM each of dNTPs, 3.33 U/μl multiscribe reverse transcriptase and 0.25 U/μl RNAse inhibitor (all purchased from cDNA Archive kit of Applied Biosystems). Real-time PCR was performed using an Applied Biosystems 7500 Fast Real-Time PCR system. The 10 μl PCR volume included 0.67 μl of RT product, 1× TaqMan universal PCR master mix and 1 μl of primers and probe mix of the Taq-Man MicroRNA assay (PE Applied Biosystems). Ct were determined using default threshold settings. miRNA let-7a was identified as the most stable housekeeping miRNA in lymphmonocytes and used to normalize the expression of each miRNA [24]. Relative quantification (RQ) of miRNA expression was calculated with the 2-ΔΔCt method (Applied Biosystems User Bulletin N°2 [P/N 4303859]). Data are presented as relative quantity (RQ) of target miRNA, normalized with respect to miR-let-7a and a calibrator sample. As a calibrator, we used CD8^+^ T cells isolated from healthy normal donors.

### Anti-miRNAs transfection

Anti-hsa-miR-198 miScript miRNA Inhibitor (MIN0000228) and Anti-hsa-miR-29b miScript miRNA Inhibitor (MIN0000100) were purchased from Qiagen (Hilden, Germany). miScript inhibitor negative control (Qiagen), which targets the sequence of AllStars negative control siRNA and has no homology to any known mammalian gene, was also used. PBMCs were seeded into 12-well plates at a density of 2 × 10^6^ cells per well in 1 mL RPMI-1640 medium (Sigma-Aldrich) containing FBS (10 %) and Glutamine (1 %). Cells were transfected with 10–50–250 nM miRNA inhibitors using Oligofectamine™ Transfection Reagent (Life Technologies), according to manufacturer’s instruction. After incubating the transfected cells for 24–48 h, western blotting analysis for JAK3 and MCL-1 protein expression and apoptosis assay were performed as described above.

### Statistical analysis

Data are reported as mean ±SD and analysed for differences using an unpaired Student’s *t* test (Statview^®^ software package, SAS Institute Inc. Cary, NC, USA). p values <0.05 were considered as significant.

## Results

### Tumor-specific CD8^+^ T cell generation from RCC patient TC and allogenic HLA-matched healthy donors

Tissue specimens were obtained from 30 patients undergoing radical or partial nephrectomy for unilateral renal cell carcinoma and primary tumor cells were established in vitro. The morphological features of the tumor tissues corresponded to well-differentiated renal cell carcinomas of the clear cell type, showing prominent cytoplasmic clearing and thin-walled vascular channels. Based on its preferred long-term in vitro growth characteristics, the TC RCC cell line was selected for prospective use as a stimulator of T cell responses in vitro. We selected two healthy donors with a complete HLA match to patient TC, with donor 2 representing a healthy sibling to patient TC. Using TC RCC cells, we stimulated autologous and two HLA-matched normal donor PBMCs in vitro in mixed lymphocyte-tumor cell cultures (MLTC) as previously reported [17, 22, 23]. On day 21 after culture initiation, CD8^+^ T cells were isolated by negative immunomagnetic cell separation and restimulated in an identical fashion (to the primary induction) for an additional 2 weeks. On day 35 of culture, responder-CD8^+^ T cells from TC patient and normal donor cultures were evaluated for gene expression profiles using microarray analysis and the data compared with that obtained from the autologous, freshly-isolated CD8^+^ T cells (i.e. day 0 of culture). The CD8^+^ T cell isolated purity was evaluated by flow cytometry and ranged from 95 to 98 % (data not shown).

### Analysis of gene expression differences between RCC patient TC and normal donor CD8^+^ T cells

**Fig. 1:**
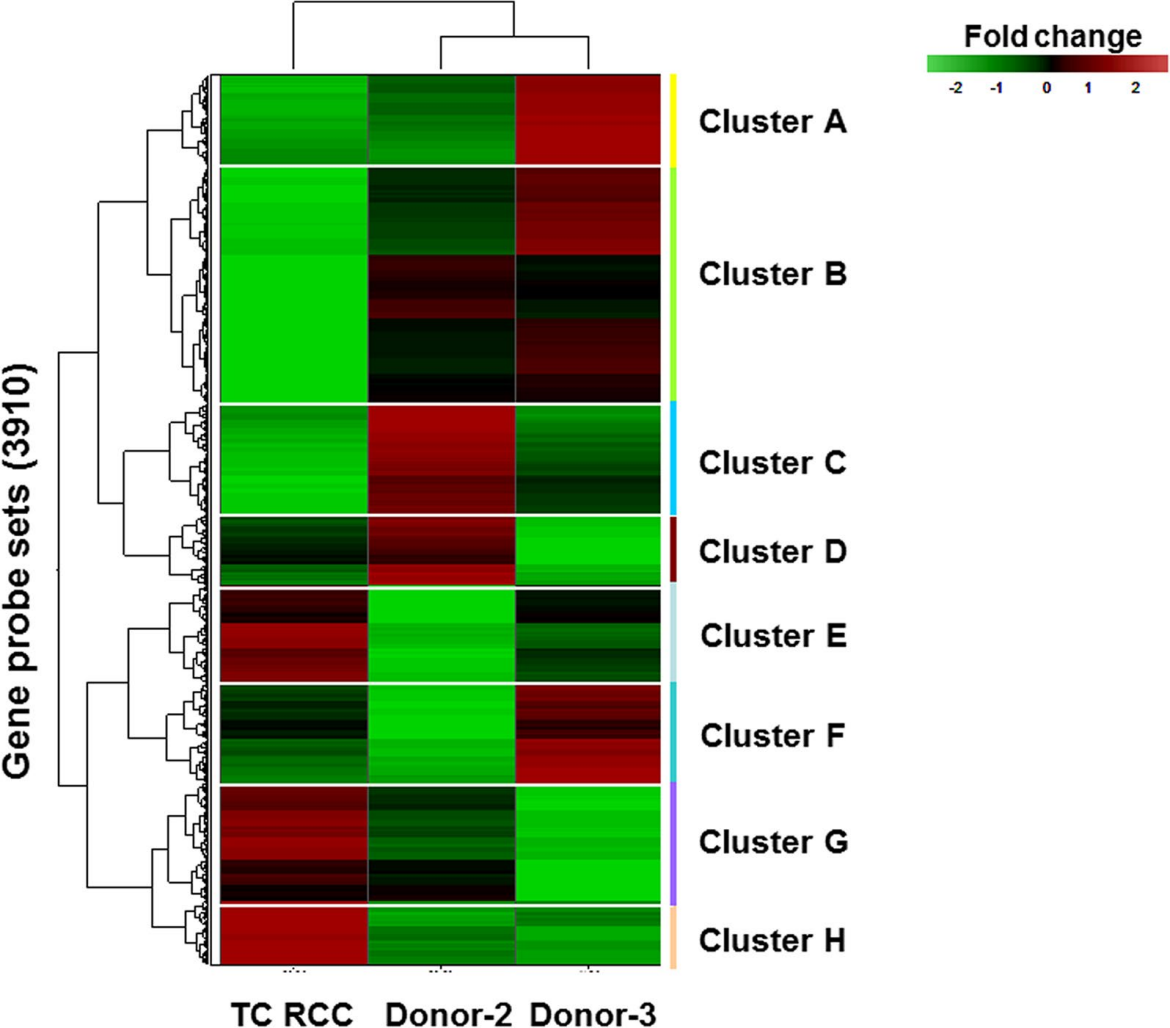
Gene expression profiling of responder CD8^+^ T cells using microarray analysis. Unsupervised hierarchical clustering of differentially expressed genes in CD8^+^ T cells isolated from RCC patient TC and 2 normal donors after 5 weeks of MLTC using RCC TC as a stimulus (day 35). Each column represents the average of normalised log intensity values for each triplicate determination. The triplicate was obtained by performing three independent MTLC stimulations in* parallel*

Gene expression profiling was performed in triplicate on patient TC and 2 normal HLA-matched healthy donors. We separately analysed at day 0 and day 35 of MLTC, CD8^+^ T cell gene expression comparing TC patient versus normal donors and selected the differentially expressed genes in both comparisons using a q-value cut-off of 0.05. We then focused on the comparison at day 35 since MLTC was able to discern a gene expression pattern distinguishing naïve and activated CD8^+^ T cells from RCC patient and HLA-matched healthy donors. At day 0, we observed only a modest modulation of those processes which were subsequently investigated at day 35. Unsupervised hierarchical clustering revealed that data derived from CD8^+^ T cells obtained from patient TC clustered differently versus CD8^+^ T cells from both normal donors (Fig. 1). Interestingly, genes included in the clusters B and H showed the best degree of discrimination between CD8^+^ T cells isolated from patient TC and the two normal donors. Genes included in cluster B (n = 391 genes) were down-regulated, while genes included in cluster H (n = 83 genes) were up-regulated compared to the healthy donors’ CD8^+^ T cells. To further study the functions of genes differentially expressed, we performed an analysis by IPA (ingenuity pathways analysis) software. Interestingly, we observed that the top molecular and cellular functions in which the differential expressed genes were included, were cell death and survival (p value range 9.46*e-11 and 8.24*e-99) and cellular growth and proliferation (p value range 9.75* e-11 and 1.57*e-85). Among the genes involved in cell death and survival, we focused on those involved in apoptosis pathway (Additional file 1: Table S1). We found a significant reduction of MCL-1 transcription factor (−1.34) in CD8^+^ T cells isolated from patient TC versus the normal donors. Additionally, we also observed that expression of the JAK3 gene product, which plays an essential role in haematopoiesis during T cell development, cell cycle and cell proliferation, was down-regulated (−1.44) in patient versus normal donor-derived CD8^+^ T cells.

### JAK3 and MCL-1 protein expression are reduced in CD8^+^ T cells isolated from RCC patient TC versus normal donors

**Fig. 2:**
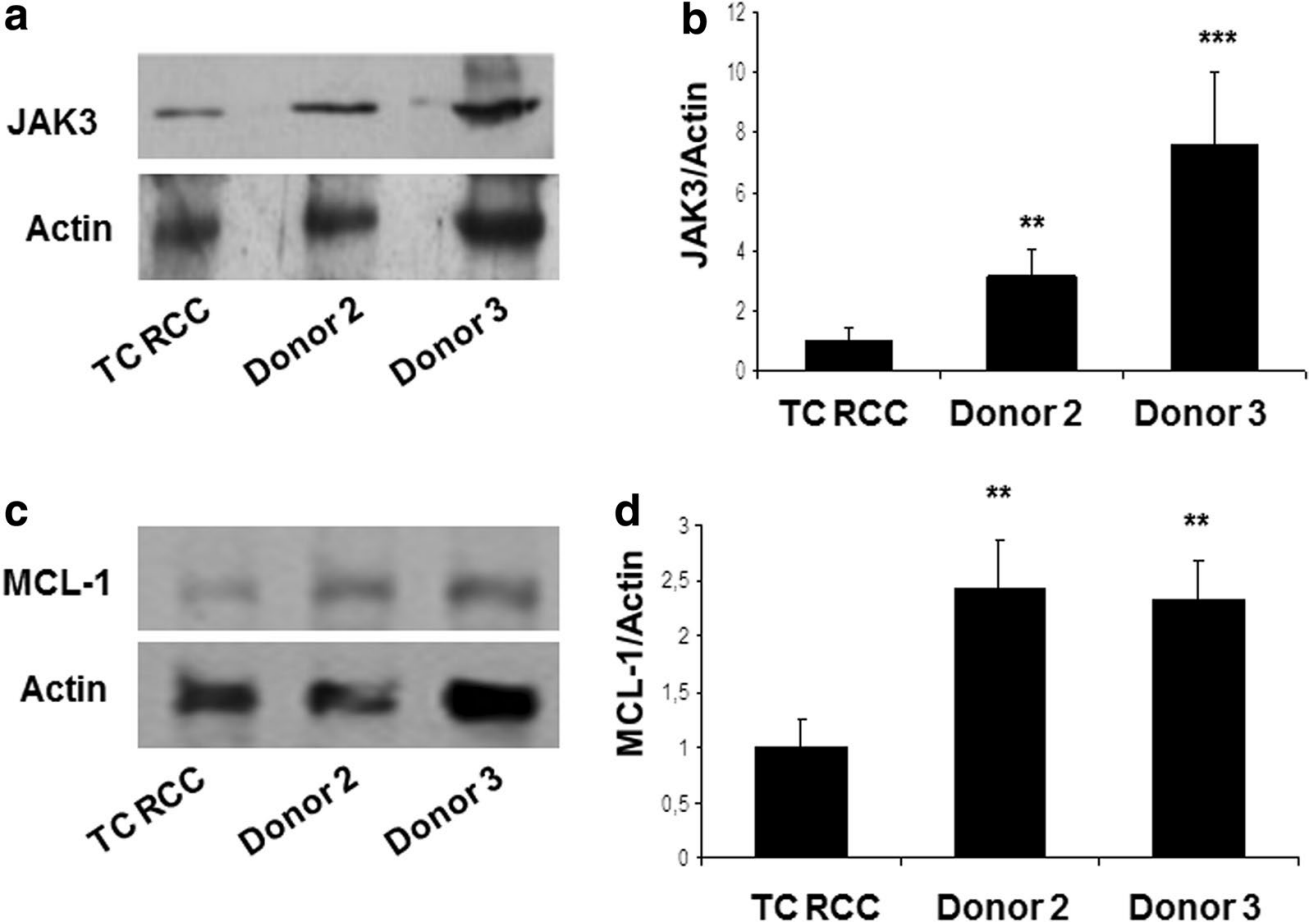
RCC-reactive CD8^+^ T cells from patient TC exhibit reduced levels of Jak3 and MCL-1 proteins. Representative immunoblots are provided for d35-cultured CD8^+^ T cells isolated from patient TC versus normal donors using specific antibodies against total Jak3 (**a**) and MCL-1 (**c**) protein. Control antibodies recognizing actin are also provided. Relative levels of Jak3 and MCL-1 (both normalized to actin) are presented in* panels* **b** and **d**, respectively. Results in* panels* **b** and **d** are reported as the mean (±SD) of ratios obtained in three independent experiments. p < 0.005 for RCC vs donor 2; p < 0.0005 for RCC vs donor 3

MCL-1 and JAK3 were selected from the list of differentially expressed genes based on their q-value, fold change and function. *MCL*-*1* is an anti-apoptotic member of the BCL-2 family, while JAK3 is known to play a crucial role in CD8^+^ T cell proliferation/differentiation [25, 26] and its expression appears deficient in CD8^+^ T cells from RCC patients, leading to T cell arrest in cell cycle progression, as we previously described [17]. Thus, we next investigated MCL-1 and JAK3 protein expression in CD8^+^ T cells isolated from MLTC cultures on day 35 using western blot analysis. Consistent with the microarray results, JAK3 and MCL-1 protein levels were down-regulated in CD8^+^ T cells isolated from the TC RCC patient when compared to normal donor controls (Fig. 2a–b versus Fig. 2c–d respectively).

**Fig. 3:**
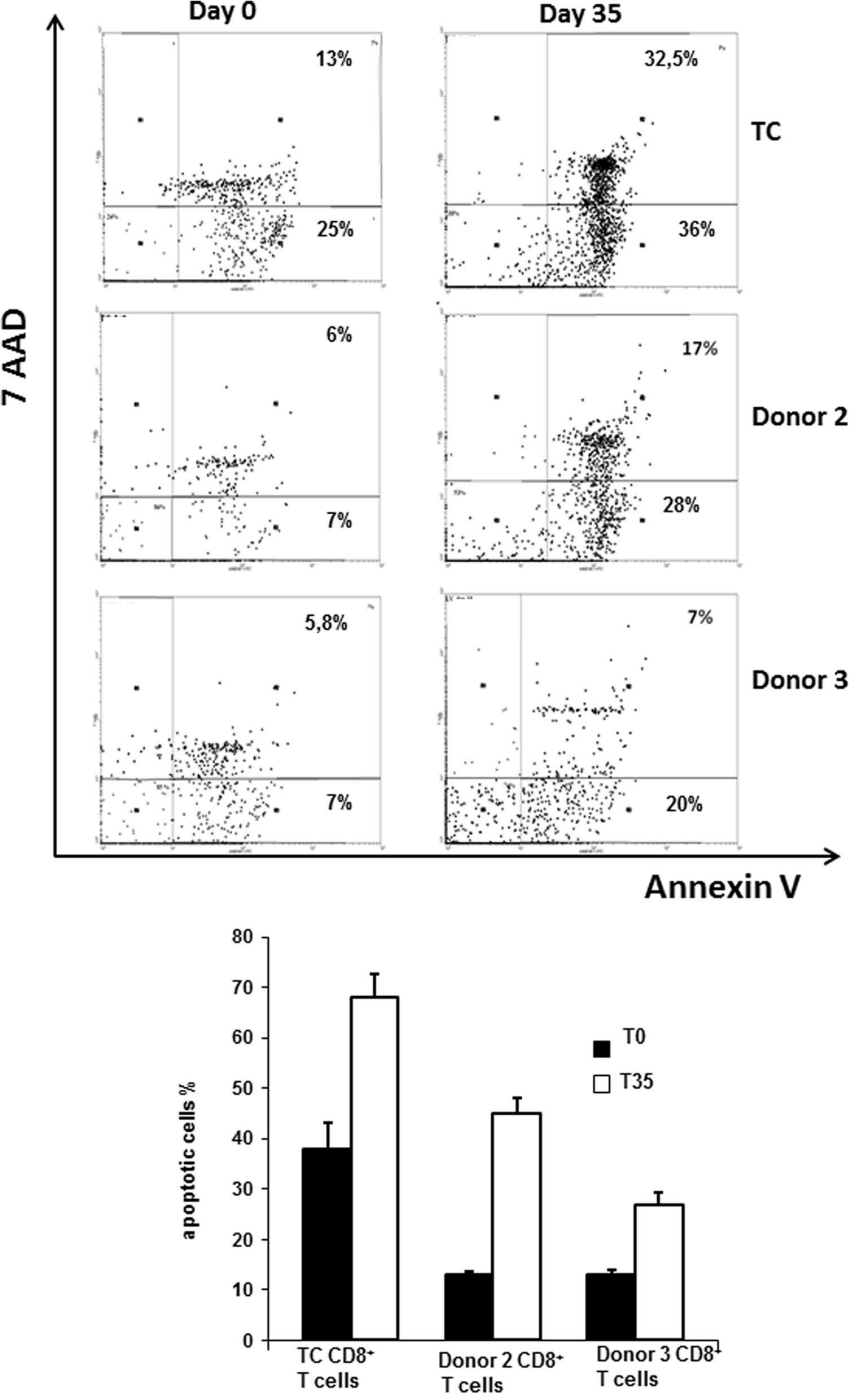
CD8^+^ T cells isolated from RCC patient TC exhibit a sustained pro-apoptotic phenotype even after extended MLTC. Expression of Annexin-V by CD8^+^ T cells isolated from patient TC or normal donors pre- and post-MLTC culture is reported. Data represent the mean ±SD percentage of apoptotic cells determined from triplicate cultures using flow cytometry. Apoptotic cells were calculated as the sum of early and late apoptotic cells. These data were reproduced in three independent experiments. At day 0, TC CD8^+^ T cells showed an increase in apoptotic cells compared to donor 2 and donor 3 (p < 0.0001 and p < 0.0001 by Student’s unpaired *t*-test). In vitro stimulation caused a slight decrease in apoptotic cell numbers in patient TC CD8^+^ T cells, but they were still higher than controls (p < 0.0035, p < 0.0001, respectively)

To evaluate the functional fate of CD8^+^ T cells isolated from RCC patient and healthy normal donors, we stained T cells for expression of Annexin-V/7-AAD and analysed these cells by flow cytometry. As shown in Fig. 3, we noted that CD8^+^ T cell from RCC patient TC on day 0 exhibited a higher apoptotic rate when compared with T cells from the two normal donors (p < 0.003) and this data was exacerbated after 35 days of MLTC (p < 0.0001).

### CD8^+^ T cells from RCC patient TC overexpress miR-29b and miR-198

**Fig. 4:**
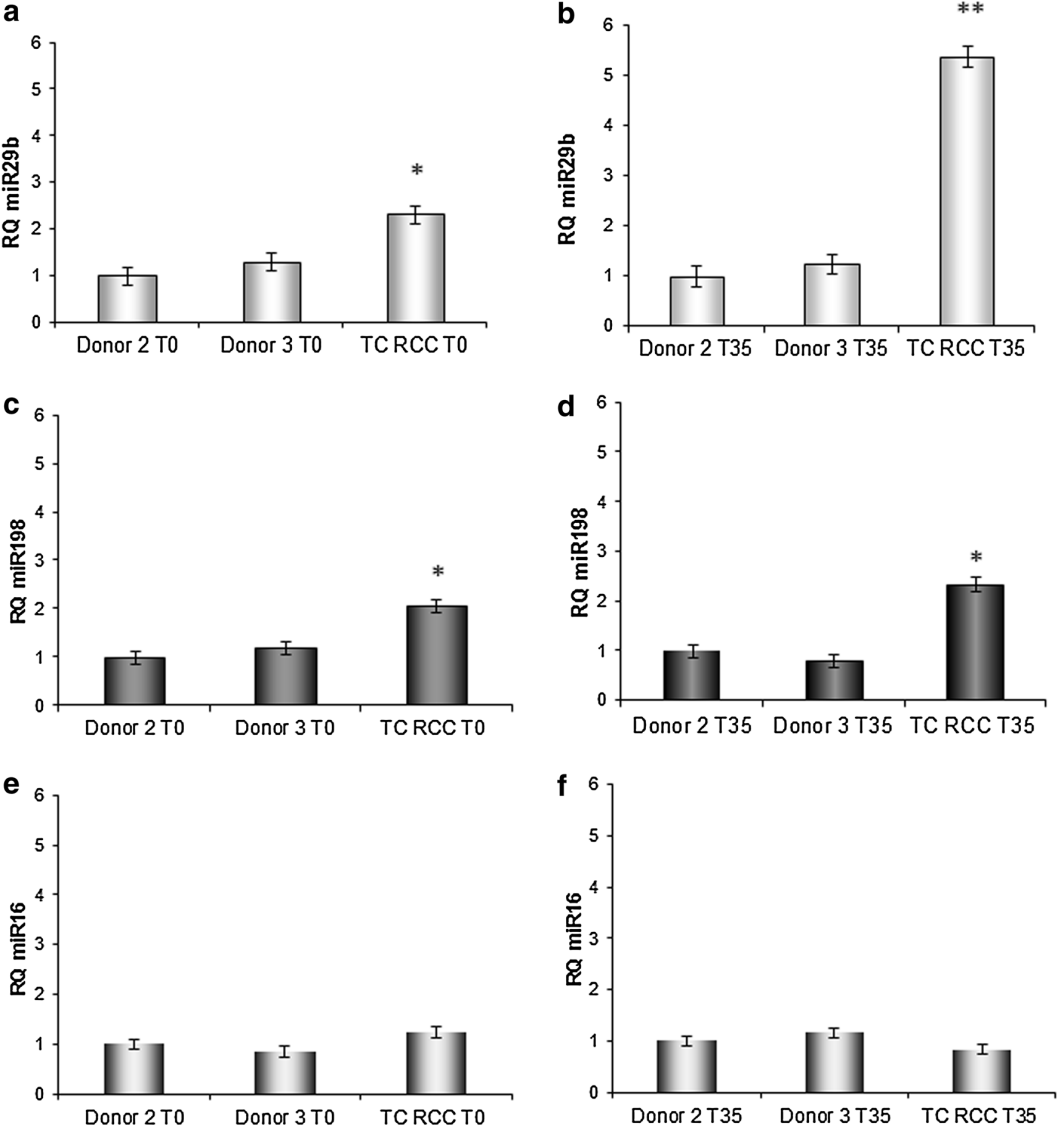
CD8^+^ T cells isolated from RCC patient TC express elevated levels of miR-29b and miR-198 when compared to normal donor T cells. Real-time PCR analysis of mir-29b (*panels* **a**, **b**) and mir-198 (*panels* **c**, **d**) expression in CD8^+^ T cells from TC RCC and both HLA-matched normal donors, at day 0 (T0;* panels* **a**, **c**) and after 35 day of stimulation (T35;* panels* **b**, **d**). *p < 0.05 for RCC patient TC versus normal donors at T0. **p < 0.005 and *p < 0.03 for RCC patient TC versus normal donors at T35. Data are representative of 3 independent experiments performed. miR16 expression in CD8^+^ T cells from TC RCC and both HLA-matched normal donors at T0 (**e**) and T35 (**f**)

In order to clarify the mechanism underlying CD8^+^ T cell dysfunction in RCC patients, we assessed whether differential expression of regulatory microRNA might influence MCL-1 and JAK3 gene expression. According to published data, we determined that miR-29b targets MCL-1 gene expression [27]. Moreover, database information (PicTar: http://pictar.mdc-berlin.de, DIANA TOOLS: http://diana.imis.athena-innovation.gr/DianaTools/index.php, PITA: http://omictools.com/pita-s973.html) suggests that miR-198 may serve as regulators of JAK3 gene expression. Our analysis on freshly-isolated CD8^+^ T cells (day 0) indicated that both miR-29b and miR-198 expression were elevated in CD8^+^ T cells isolated from patient TC (Fig. 4a, b; p < 0.05 versus either normal donor). After 35 days of MLTC stimulation, patient T cell overexpression of miR-29b (Fig. 4c; p < 0.005 versus either normal donor) and miR-198 (Fig. 4d; p < 0.03 versus either normal donors) was maintained, and that of miR-29b was further enhanced. miR16 was chosen as negative control miRNA, with no modulation of its expression observed between RCC patient and healthy donors (Fig. 4e, f).

### Peripheral blood CD8^+^ T cells from RCC patients display elevated expression of miR-29b and miR-198

**Fig. 5:**
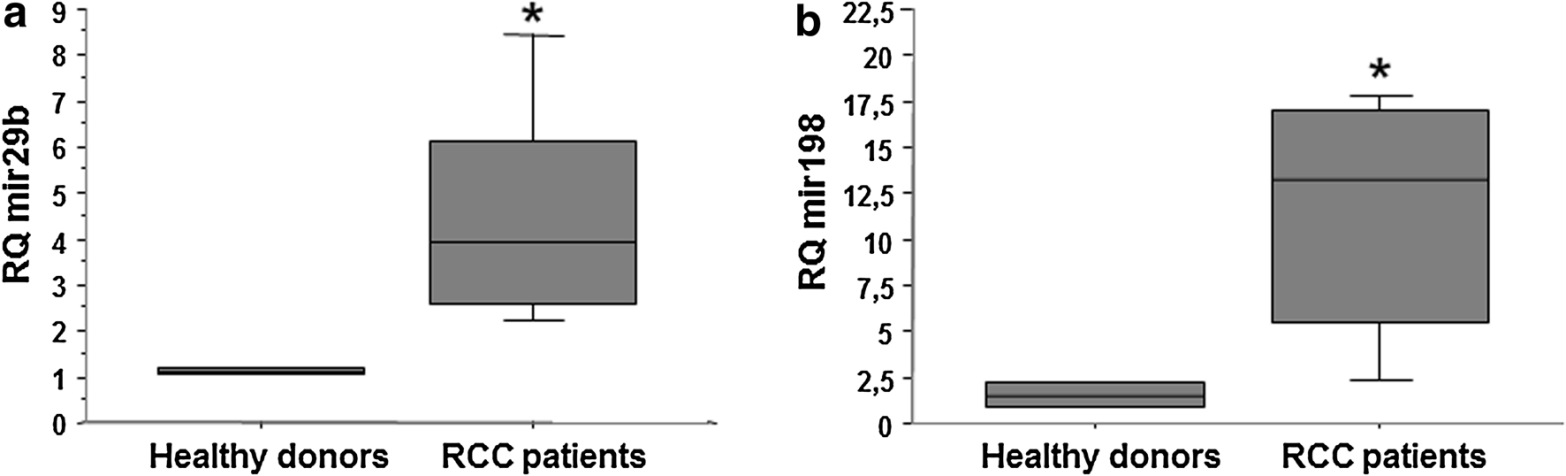
Freshly-isolated CD8^+^ T cells from RCC patients exhibit elevated levels of miR-29b and miR-198 versus normal donors. CD8^+^ T cells were MACS-isolated from the PBMC of 25 primary RCC patients and 15 healthy normal donors. After extraction of RNA, quantitative RT-PCR was performed for Mir-29b (panel **a**) and Mir-198 (*panel* **b**) as described in "Methods" section. Data are presented in box plots; *p < 0.05 for RCC patients versus healthy normal donors

The results obtained for RCC patient TC prompted us to investigate the generality of miR-29b and miR-198 overexpression in a larger cohort of RCC patients (Table 2). CD8^+^ T cells were freshly isolated from the peripheral blood of 25 RCC patients and age matched-15 healthy normal donors. As shown in Fig. 5 (panels a and b), expression levels of both miR-29b and miR-198 were significantly up-regulated in RCC patients (p < 0.02 and p < 0.01 respectively) when compared with normal donors. Furthermore, to evaluate whether the upregulation of miR29b and miR198 in RCC patients was correlated to decreased MCL-1 and JAK3 gene levels, we monitored mRNA expression levels by qRT-PCR. As expected, we found that MCL-1 gene expression was significantly lower in RCC patients compared to healthy subjects (p < 0.0001; Additional file 2: Figure S2). Similarly, JAK3 gene expression was downregulated in RCC patients compared to controls but not so pronounced as MCL-1 trend (p < 0.005).

**Table 2:** RCC patient and healthy donor demographics

Patient	Age/sex	Stage	Grade	Adjuvant treatment	Healthy donor	Age/sex
1	42 F	T3aN0M0	2	–	1	41 M
2	53 M	T1N0M0	3	–	2	50 M
3	60 M	T3aN0M0	2	–	3	54 M
4	54 F	T3aN0M0	2	–	4	52 F
5	68 M	T3aN0M0	3	–	5	67 M
6	65 F	T3aN0M0	3	–	6	60 F
7	65 F	T3bN0M0	1	–	7	61 F
8	54 F	T3aN0M0	3	–	8	50 F
9	67 M	T3aN0M0	2	–	9	58 M
10	74 M	T3bN0M0	2	–	10	72 M
11	53 M	T1bN0M0	1	–	11	51 M
12	57 M	T1aN0M0	2	–	12	57 M
13	63 F	T1N0M0	1	–	13	53 F
14	80 F	T1bN0M0	2	–	14	72 F
15	65 F	T1aN0M0	2	–	15	61 F
16	72 M	T1N1M0	2	–		
17	64 F	T2N1M0	2	–		
18	43 M	T1aN1M0	3	–		
19	62 M	T1bN0M0	1	–		
20	33 M	T3bN0M0	2	–		
21	69 M	T1aN0M0	2	–		
22	72 M	T3bN0M0	2	–		
23	53 F	T2N0M0	2	–		
24	61 F	T3aN0M0	3	–		
25	60 M	T3bN0M0	2	–		

RCC patients and healthy donors evaluated in the study. For RCC patients were considered age at time of surgery, sex, pathological stage, grade (Fuhrman), adjuvant treatment and at the last evaluation, follow-up months

### Inhibition of miR-29b and miR-198 enhance the expression of MCL-1 and JAK3 and protects RCC PBMCs from apoptosis

**Fig. 6:**
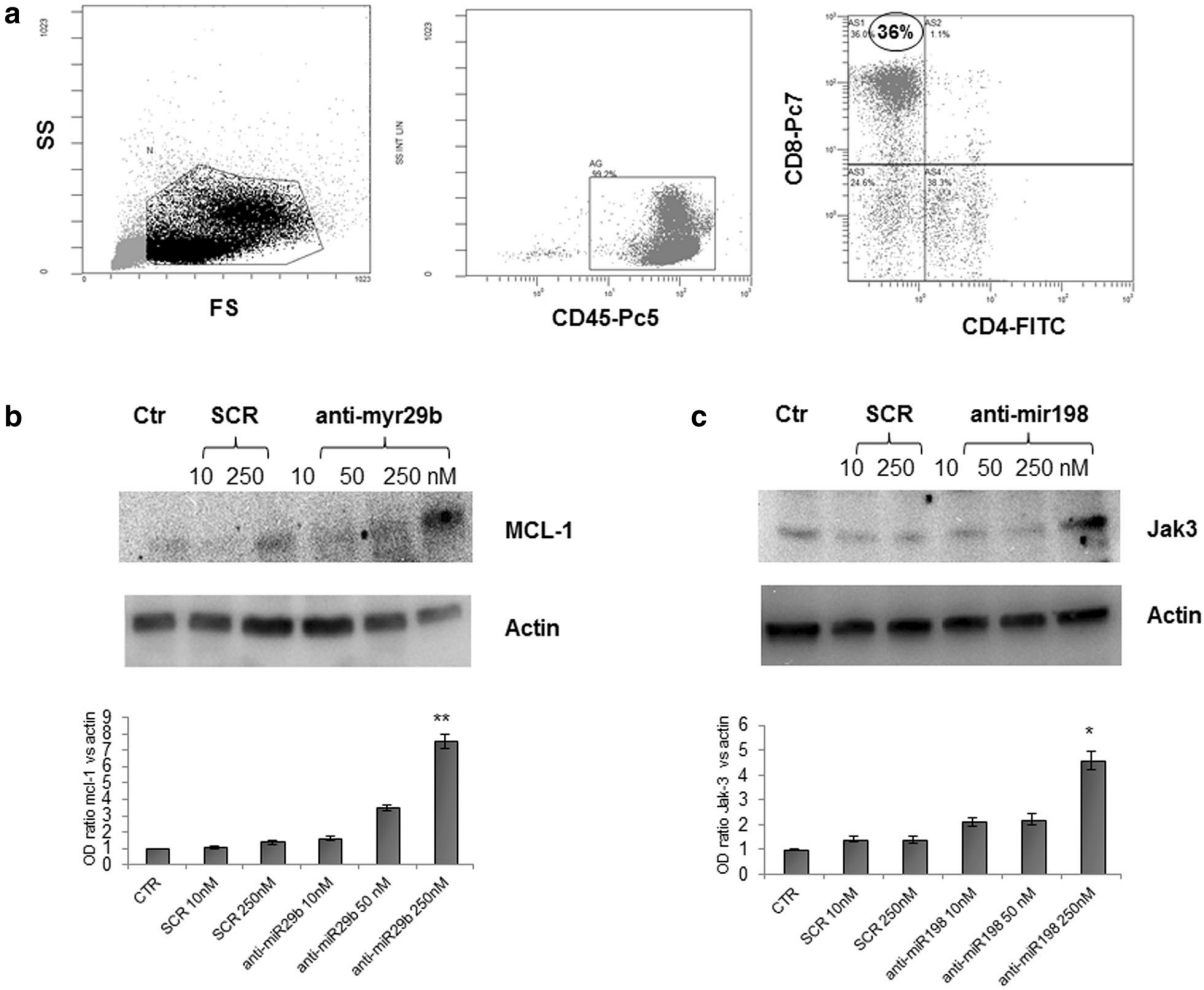
Transfection with specific Anti-hsa-miR inhibitors upregulate MCL-1 and JAK3 protein expression and rescues the cells from apoptosis. The percentage of CD8^+^ T cells in a freshly isolated PBMC sample is depicted from one RCC patient (**a**). PBMCs from RCC patients were transfected with 10, 50 and 250 nM Anti-hsa-miR-29b or Anti-hsa-miR-198 inhibitor and 10 and 250 nM scrambled miR control (SCR), as outlined in “Methods” section. After 48 h cell lysates were generated and western blots performed to quantitate MCL-1 (**b**) and JAK3 (**c**) protein levels. Three independent experiments were performed and gels showing MCL-1 and JAK3 protein levels were scanned and quantified. **p < 0.005 and *p < 0.01 versus cells only treated with 250 nM scrambled miR control

To validate that miR-29b and miR-198 negatively regulate protein expression of MCL-1 and JAK3 in T cells, transfection experiments were performed using their specific miRNA inhibitors: Anti-hsa-miR-29b and Anti-hsa-miR-198. Given the high mortality rate observed after transfection of isolated CD8^+^ T cells, we decided to transfect total PBMCs and, as showed in Fig. 6 (panel a), the percentage of CD8^+^ T-lymphocytes ranged between 30 and 45 % in PBMC samples from selected RCC patients. We selected those patients which expressed high miR-29b and miR-198 levels and freshly isolated PBMCs were transfected using Oligofectamine™ Transfection Reagent with both Anti-hsa-miRNAs. As shown in Fig. 6 (panels b and c), silencing of miR-29b and miR-198 for 48 h at 250 nM (no effects were observed at 24 h and at 10 and 50 nM), resulted in a marked induction of both protein levels when compared with scrambled miR control (p < 0.005 and p < 0.01, respectively).

**Fig. 7:**
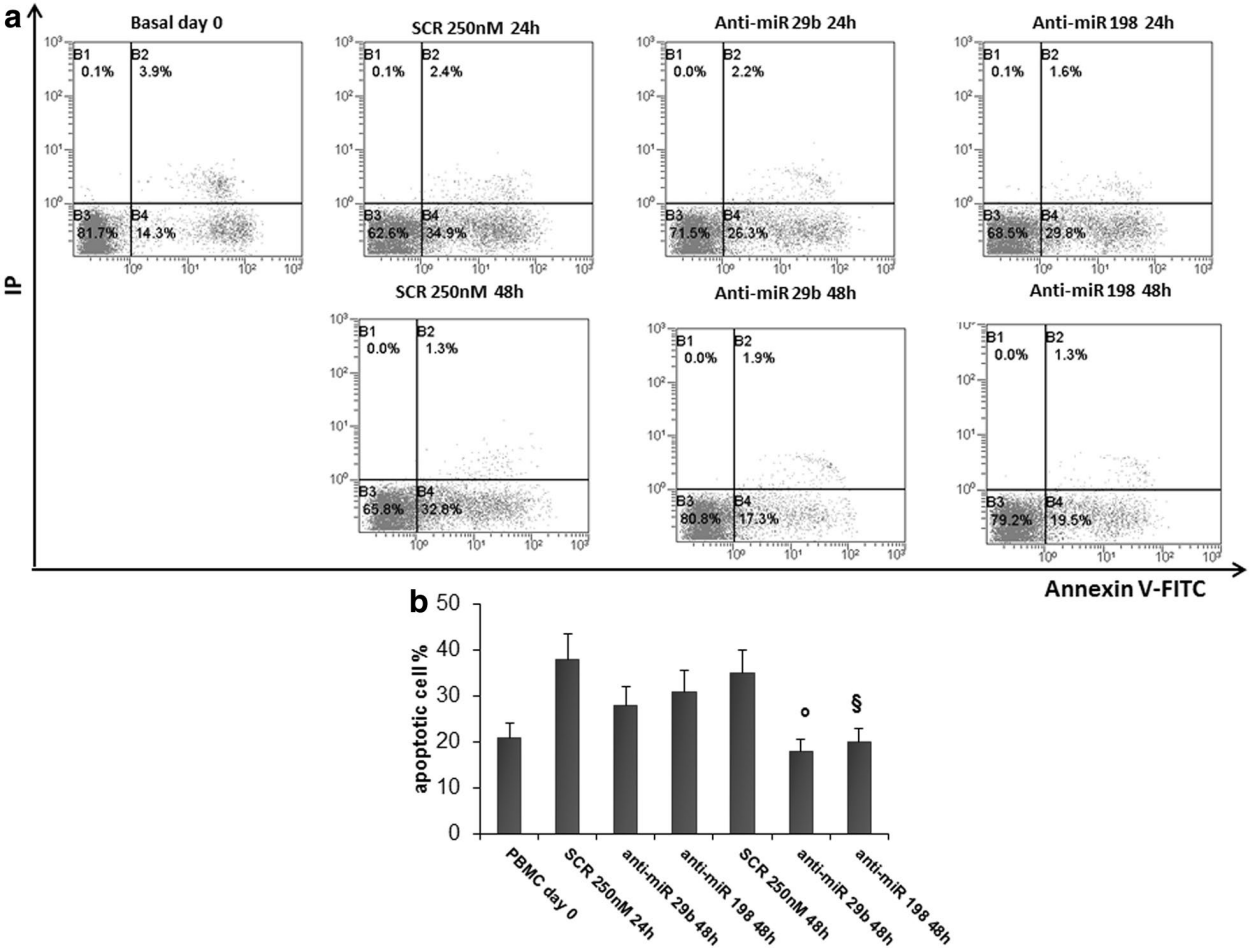
Transfection with specific Anti-hsa-miR inhibitors upregulate MCL-1 and JAK3 protein expression and rescues the cells from apoptosis. Annexin V/PI assays in PBMCs after 48 h of transfection with Anti-hsa-miR-198 or Anti-hsa-miR-29b. Apoptotic cells were calculated as the sum of early and late apoptotic cells (**a**). The results are shown as percentage of apoptotic cells. Data are the average of six independent experiments ±SD, p < 0.01 and §p < 0.03 versus SCR (**b**)

To directly address miR-29b and miR-198 specificity and MCL-1 and JAK3′s involvement in tumor suppressor activity, we investigated the effect of both miRNAs inhibition on apoptosis, as induction of immune cell apoptosis is a crucial event during malignant transformation. Interestingly, as shown in Fig. 7 (panels a and b), we found that 48 h transfection with Anti-hsa-miRNA29b and Anti-hsa-miRNA198, rescued the cells from cell death and there was a significant reduction of cell apoptosis when compared with cells transfected with scrambled miR control, as measured by the Annexin V assay (p < 0.01 and p < 0.03, respectively).

## Discussion

Over the past few years, the discovery of miRNAs has changed the landscape of human genetics and they have been widely studied in the field of oncology, where altered miRNA expression has been observed in a variety of human cancers [28]. Indeed, miRNA-expression profiling of human epithelial malignancies has led to the identification of signatures associated with diagnosis, staging, progression, prognosis and even response to treatment [29, 30]. However, few mechanistic studies have been carried out to dissect the functional role of miRNA in RCC, particularly in the context of “immune dysfunction” which may underlie disease progression. In fact, attaining effective tumor immunity is a major goal of the modern biologic therapy, limited by the tumor microenvironment and regulatory mechanisms affecting T cell and NK cell effectors [31].

The major finding in this report is that CD8^+^ T cells isolated from RCC patients exhibit defects in proliferation and a propensity to undergo apoptotic cell death as a consequence of reduced expression of *JAK3* and MCL-1, the expression of which are regulated in vivo by tumor-induced upregulation of miRNAs miR198 and miR29b, respectively. To probe the clinical relevance of this hypothetical defect in antitumor T cell dysfunction amongst RCC patients, we investigated the genomic profile of tumor-reactive CD8^+^ T cells obtained after extended rounds of MLTC stimulation with an highly immunogenic RCC cell line (patient TC) and compared with their HLA-matched healthy donors (donor 2 and 3). Our results indicate that CD8^+^ T cells from patient TC cluster differently from normal donors and many of the genes differentially expressed were implicated in critical pathways, such as apoptosis, cell cycling and proliferation. On the basis of the microarray data, we chose to focus on MCL-1 and JAK3 genes which appeared downregulated in tumor-reactive CD8^+^ T cells from RCC patient. *JAK3* is a cytoplasmic tyrosine kinase and plays critical roles in cytokine signaling for development, differentiation, proliferation and survival of T-cells. Deregulation in JAK/STAT interaction promote tumorigenesis in haematological malignancies and epithelial tumors representing new therapeutic target [32, 33] and in our report we showed JAK3 mutations are associated with metastatic spread and poor survival of RCC patients [21]. MCL-1 is an anti-apoptotic member of the BCL-2 family that is composed of both pro-apoptotic and anti-apoptotic proteins, and the balance between these proteins seems to determine life or death for the cell [34]. Bcl-2, MCL-1, and Bcl-Xl are anti-apoptotic proteins and promote cell survival and proliferation. Aberrant MCL-1 overexpression in numerous human cancers has been shown to result in increased proliferation, tumor cell survival, drug resistance and metastasis [35, 36]. On the other hand, coordinate down-regulation of MCL-1 and JAK3 in T cells from RCC patients could represent a mechanism of immune evasion which confers a survival advantage to the tumor in the face of a muted attack by the immune system.

miRNAs regulate pro- and anti-apoptotic genes involved in programmed cell death pathways [37] and also play an important role in anti-tumor immunity by regulating the differentiation program of cytotoxic T lymphocytes (CTLs) [38]. The relevance of microRNAs in T cell immunity is highlighted by the finding that the lack of Dicer (a key enzyme for miRNA biogenesis) expression in CD8^+^ T cells results in impaired effector function(s) and differentiation [39]. Furthermore, a limited set of miRNA appears to regulate the differentiation program of CD8^+^ T cells following the priming of naïve cells into effector memory cells [40, 41]. However, the role of individual miRNAs in the CD8^+^ T cell response has been only poorly explored to date. In the present study, we determined that MCL-1 and JAK3 expression levels in T cells are counter-regulated by miR-29b and miR-198, respectively. miR-29b has been shown to be amplified in various tumor types including myeloid leukemia [42, 43] and hepatocellular carcinoma [44], serving to promote tumorigenesis. This is the first report that investigates the expression and function of miR-29b and miR-198 in T cells from RCC patients. We observed that the high expression levels of these two miRNAs in CD8^+^ T cells could clearly discriminate CD8^+^ T cells freshly harvested from RCC patients versus normal donors. Notably, these molecular defects were retained in RCC patient TC even after extended rounds of MLTC stimulation, suggesting the need for sustained, targeted intervention if complete “normalization” of the RCC patient anti-tumor CD8^+^ T effector cell repertoire is desired as a (clinical) endpoint. Proof-of-principle may be suggested by our ability to resurrect MCL-1 and *JAK3* expression in PBMCs from RCC patients upon transfection with anti-hsa-miR-29b and anti-hsa-miR-198 inhibitors. Our data confirm, as already demonstrated, that MCL-1 is a direct target of miR29b [27] and support the hypothesis that miR-198 directly or indirectly regulates JAK3 expression. Interestingly, the silencing of both miRNAs for 48 h resulted in improvement of cell survival and protection from apoptosis.

It remains unclear what environmental cues provided by the tumor microenvironment in vivo or under conditions of co-culture with tumor cells in vitro serve to sustain elevated levels of regulatory miRs such as miR29b and miR198 in RCC patient T cells. Conversely, will therapies known to enhance the anti-tumor immunity (i.e. IFN-alpha, sunitinib, etc.) suppress expression of these miRs in patient T cells in vivo? These are major questions that warrant prospective studies in order to further elucidate and counter—act the pro-tumor impact of miRs on protective host immunity, thereby optimizing the therapeutic potential of anti-tumor CD8^+^ T effector cells.

## Conclusion

CD8^+^ T cells isolated from RCC patients exhibit defects in proliferation and a propensity to undergo apoptotic cell death as a consequence of reduced expression of JAK3 and MCL-1 that are regulated in vivo by overexpressed miRNAs miR198 and miR29b, respectively.

## Additional files

10.1186/s12967-016-0841-9 Down-regulated Genes in apoptosis dataset.
10.1186/s12967-016-0841-9CD8^+^ T cells from RCC patients exhibit downregulated MCL-1 and JAK3 gene expression. Real-time PCR analysis of MCL1 and JAK3 expression in freshly-isolated CD8^+^ T cells from RCC patients and healthy normal donors. *** p < 0.0001 and ** p < 0.005 for RCC patients versus healthy normal donors.
