# Supplementary material for: miR-29b and miR-198 overexpression in CD8+ T cells of renal cell carcinoma patients down-modulates JAK3 and MCL-1 leading to immune dysfunction
Source: J Transl Med. 2016 Apr 11;14:84. doi: 10.1186/s12967-016-0841-9 (PMC4827202; doi:10.1186/s12967-016-0841-9)
Supplement: Supplementary file 1 — 10.1186/s12967-016-0841-9 Down-regulated Genes in apoptosis dataset. [file 12967_2016_841_MOESM1_ESM.pdf]

Table S1

Down-regulated Genes in apoptosis dataset

Genes	Exp Fold Change
ABCA1	-1.362
ABCC1	-1.492
ABCE1	-1.304
ABL1	-1.337
ACLY	-1.361
ACSL4	-1.54
ADAMTS1	-1.469
ADI1	-1.371
ADORA2B	-1.45
ADRBK1	-1.659
AIF1	-1.344
AIFM1	-1.34
AIM2	-1.888
ALDOA	-1.328
ANG	-1.345
ANP32A	-1.329
ANPEP	-1.371
ANXA6	-1.448
API5	-1.318
APOBEC3B	-4.661
AREG	-2.274
ARG2	-1.811
ARHGDIA	-1.649
ARID3B	-1.331
ARNT	-1.325
ARRB1	-1.321
ARRB2	-1.41
ATAD2	-3.018
ATF2	-1.47
ATG7	-1.304
ATP2C1	-1.419
ATP7A	-1.304
ATXN3	-1.315
AURKA	-2.785
AURKB	-2.792
B4GALT5	-1.377
BAG3	-1.442
BCL10	-1.833

Genes	Exp Fold Change
BCL2	-1.368
BHLHE40	-2.184
BIN1	-1.303
BIRC5	-1.86
BLM	-1.352
BLVRA	-1.354
BMI1	-1.484
BNIP1	-1.375
BNIP3	-1.323
BRCA1	-2.842
BRCA2	-1.35
BSG	-1.855
BUB1	-1.88
C1QBP	-1.686
CADM1	-1.393
CALR	-1.384
CANX	-1.344
CAPN1	-1.41
CAPN3	-1.332
CAPNS1	-1.319
CASP3	-1.456
CASP8	-1.468
CASP9	-1.851
CAST	-1.383
CBFB	-1.737
CBX4	-1.538
CBX5	-1.362
CCNA1	-1.396
CCNA2	-2.235
CCNB1	-4.159
CCNC	-1.498
CCND2	-1.929
CCND3	-1.545
CCR7	-6.022
CCR8	-1.928
CCT2	-1.638
CCT4	-1.464
CD164	-1.301
CD28	-1.376
CD33	-1.56
CD38	-1.398

Genes	Exp Fold Change
CD5	-1.617
CD55	-1.659
CD7	-2.099
CD70	-1.618
CD74	-1.621
CD80	-1.594
CD9	-1.563
CDC20	-3.357
CDC25A	-1.757
CDC25C	-1.386
CDC37	-1.498
CDC42	-1.656
CDC45	-1.901
CDC6	-2.628
CDC7	-1.512
CDK1	-2.605
CDK19	-1.37
CDK2	-1.438
CDK4	-1.783
CDKN2C	-1.474
CDKN3	-2.55
CEBPD	-1.338
CENPA	-1.374
CENPE	-2.31
CENPF	-1.716
CENPJ	-1.403
CFH	-1.363
CFLAR	-1.368
CHEK1	-2.457
CHEK2	-1.387
CIT	-2.247
CITED2	-1.711
CKAP2	-1.623
CKAP5	-1.485
CLASP1	-1.564
CLIC4	-1.361
COL18A1	-1.309
COMMD3-BMI1	-1.484
CORO1A	-1.607
COX8A	-1.317
CREB1	-1.35

Genes	Exp Fold Change
CREM	-1.386
CSE1L	-1.502
CSNK2A1	-1.426
CTBP1	-1.457
CTH	-1.507
CTSH	-1.388
CUL4B	-1.608
CUL5	-1.345
CXCL2	-1.769
CXCL8	-1.311
CXCR3	-1.313
CXCR4	-1.392
CYCS	-1.398
CYP1B1	-1.387
DAPK3	-1.378
DCAF7	-1.327
DDX11	-1.319
DEDD	-1.302
DEPDC1	-1.591
DESI2	-1.36
DHCR24	-1.456
DHFR	-1.496
DHX9	-1.437
DMC1	-1.448
DNAJC15	-1.372
DNM2	-1.535
DNMT1	-1.567
DTYMK	-1.583
DUSP10	-1.494
DUSP4	-1.653
DUT	-1.342
E2F8	-2.774
EAF2	-1.674
ECT2	-2.292
EGR3	-1.364
EHD4	-1.602
EHMT2	-1.406
EI24	-1.327
EIF2AK2	-1.404
EIF4B	-1.51
EIF4E	-1.759

Genes	Exp Fold Change
EIF5A	-2.619
ELAVL1	-1.324
ELK1	-1.324
ELMO1	-1.424
ELOVL4	-1.403
ENO1	-1.701
ENTPD1	-1.465
EPAS1	-1.392
ESPL1	-1.927
ETS2	-1.391
ETV5	-1.308
EXO1	-1.853
EZR	-1.389
FAF1	-1.542
FAH	-1.406
FAIM	-1.868
FAM162A	-1.387
FANCA	-1.405
FASN	-1.366
FBL	-1.579
FBXO5	-2.186
FCGR3A/FCGR3B	-1.589
FEM1B	-1.344
FEN1	-2.101
FES	-1.406
FGR	-1.94
FKBP1A	-1.791
FLI1	-1.81
FLNA	-1.636
FLNB	-1.43
FLOT2	-1.516
FLT3LG	-2.093
FN1	-1.683
FOS	-1.633
FOSB	-1.303
FXN	-1.705
GABBR1	-1.888
GADD45B	-1.514
GALNT10	-1.539
GAPDH	-1.313
GEM	-1.454

Genes	Exp Fold Change
GFPT1	-1.645
GINS1	-3.383
GLS	-1.315
GLUD1	-1.425
GMDS	-1.637
GMFB	-1.397
GMNN	-1.503
GNA13	-1.381
GNAI2	-1.683
GNB2	-1.398
GNLY	-2.431
GPR132	-1.52
GPR18	-1.33
GPX4	-1.377
GRB10	-1.591
GRN	-1.537
GSR	-1.444
GSTP1	-1.446
H2AFX	-2.316
HELLS	-2.359
HIPK1	-1.347
HIPK3	-1.356
HLA-DMA	-1.31
HLA-G	-1.425
HLF	-1.328
HLTF	-1.402
HMGA1	-1.498
HMGB1	-1.332
HMGN5	-1.476
HMMR	-3.539
HNRNPA1	-1.461
HNRNPC	-1.3
HPSE	-1.332
HSP90AA1	-1.41
HSP90AB1	-1.594
HSP90B1	-1.341
HSPA2	-1.579
HSPA4	-1.32
HSPD1	-1.811
HSPE1	-1.355
HTR2B	-1.301

Genes	Exp Fold Change
HUS1	-1.524
HUWE1	-1.311
ICMT	-1.464
IER3	-1.397
IFT57	-1.708
IGF1	-1.337
IGF2R	-1.539
IL13	-2.817
IL15	-1.41
IL1A	-2.44
IL1B	-1.36
IL2RA	-1.9
IL5	-2.689
ILF3	-1.348
ING3	-1.467
INPP4A	-1.501
INVS	-1.461
IRF4	-2.01
IRF8	-1.469
ITCH	-1.449
ITGA4	-1.526
ITGAM	-1.702
ITGB1	-1.322
ITGB3BP	-1.337
ITPR1	-1.499
IVNS1ABP	-1.421
JAK2	-1.431
**JAK3**	**-1.444**
JUND	-1.814
KAT5	-1.313
KAT7	-1.514
KHDRBS1	-1.466
KIAA0101	-2.801
KIF11	-2.56
KIF14	-2.946
KIF18A	-2.309
KIF3A	-1.472
KIR2DL1/KIR2DL3	-1.417
KIR3DL1	-1.409
KIT	-3.066
KLF10	-1.555

Genes	Exp Fold Change
KLF11	-1.943
KLF2	-1.684
KLF4	-1.604
KLHL20	-1.417
KMT2A	-1.365
KPNA2	-1.948
LANCL1	-1.308
LDLR	-1.454
LEF1	-1.442
LEPR	-1.775
LGALS1	-1.553
LGALS8	-1.317
LIF	-1.652
LIG1	-1.497
LIN7C	-1.444
LMNA	-1.379
LMNB1	-2.311
LMO4	-1.617
LSP1	-1.328
LTA	-1.796
LYN	-1.534
LYPLA1	-1.358
MAD2L1	-2.091
MALT1	-1.319
MAN2C1	-1.529
MAP2K2	-1.391
MAP2K3	-1.43
MAP2K4	-1.327
MAP3K1	-1.367
MAPK13	-1.423
MAPK14	-1.646
MAPKAP1	-1.326
MAPKAPK2	-1.49
MBD4	-1.412
**MCL1**	**-1.342**
MCM10	-2.753
MCM2	-3.256
MCTS1	-1.393
MDH1	-1.398
MEF2A	-1.608
MELK	-1.974

Genes	Exp Fold Change
MIF	-1.351
MIS18A	-1.56
MKI67	-2.63
MMP14	-1.371
MOB1A	-1.667
MRE11A	-1.642
MRPS30	-1.485
MS4A1	-1.428
MSH2	-1.695
MSH5	-1.755
MSH6	-1.377
MSRB2	-1.401
MYB	-6.222
MYBL1	-3.504
MYBL2	-1.564
MYO6	-1.308
NAA15	-1.552
NAA35	-1.538
NAMPT	-1.343
NBN	-1.354
NCAPG2	-3.183
NCF2	-1.337
NCOA2	-1.454
NDUFV2	-1.313
NEDD9	-1.307
NET1	-1.867
NFYA	-1.385
NME1	-1.774
NME4	-1.402
NQO1	-1.611
NR3C2	-1.561
NR4A3	-1.65
NRAS	-1.931
NT5E	-2.196
NTRK2	-1.462
NUMB	-1.312
NUP62	-1.557
NUSAP1	-1.898
OGFOD1	-1.402
OPA1	-1.612
OSM	-1.658

Genes	Exp Fold Change
P2RX4	-1.391
P4HB	-1.977
PA2G4	-1.542
PAK1	-1.539
PAK2	-1.482
PBK	-3.638
PCNA	-1.941
PCTP	-1.304
PDE4D	-1.311
PDGFD	-1.801
PECAM1	-1.473
PERP	-1.999
PF4	-1.325
PHB	-2.353
PIK3CB	-1.322
PIK3CG	-1.732
PIK3R2	-1.482
PKN2	-1.371
PLA2G16	-1.357
PLA2G4A	-1.682
PLAC8	-2.208
PLAGL1	-1.341
PLK1	-2.018
PLK4	-1.874
PMAIP1	-1.533
PML	-1.609
PMS2	-1.587
POLR2A	-1.341
PON2	-1.446
PPARG	-1.809
PPID	-1.648
PPM1D	-1.319
PPP1CA	-1.416
PPP2R1B	-1.812
PPP2R2B	-1.935
PPP5C	-1.467
PPT1	-1.336
PRDX3	-1.483
PRDX4	-1.519
PRF1	-1.607
PRKAB1	-1.337

Genes	Exp Fold Change	Genes	Exp Fold Change
PRKAR1A	-1.893	RLN2	-1.576
PRKCD	-1.374	RNF130	-1.805
PRKD3	-1.405	RPLP0	-1.323
PRKDC	-1.539	RRAS2	-1.379
PRMT1	-1.648	RRBP1	-1.591
PRMT5	-1.398	RRM1	-2.034
PSAP	-1.514	RRM2	-4.902
PSEN1	-1.494	RRN3	-1.324
PSMB8	-1.503	RSL1D1	-1.44
PTGER4	-1.54	RTN4	-1.488
PTGIS	-2.163	RUNX3	-1.447
PTK2B	-1.706	RUVBL2	-1.426
PTK6	-1.339	RXRA	-1.344
PTPN11	-1.623	RYBP	-1.317
PTPN13	-1.31	S100A8	-1.876
PTTG1	-1.58	S1PR1	-2.621
PXN	-1.763	S1PR4	-1.334
QKI	-1.341	SAR1A	-1.46
RAB27A	-1.377	SCD	-1.303
RABGGTB	-1.363	SDC4	-1.743
RAC2	-1.683	SDHC	-1.589
RACGAP1	-2.109	SELL	-1.716
RAD21	-1.391	SERPINA1	-1.343
RAD23B	-1.362	SERPINB9	-1.347
RAD50	-1.3	SERPINE2	-1.817
RAD51	-2.833	SERPINI1	-1.385
RALB	-1.341	SET	-1.337
RANBP1	-1.632	SGK1	-2.521
RASA1	-1.383	SGMS1	-1.463
RASA4	-1.573	SGPP1	-1.455
RB1	-1.511	SHC1	-2.028
RBBP4	-1.333	SIAH1	-1.624
RBL1	-1.514	SIRT1	-1.42
RBM25	-1.351	SKP2	-1.864
RDX	-1.41	SLC25A6	-1.474
REL	-1.713	SLC2A1	-1.549
RELA	-1.519	SLC2A3	-1.717
RFC1	-1.331	SLC6A6	-1.607
RGS3	-1.38	SMAD2	-1.447
RHOB	-1.841	SMAD7	-1.818
RHOC	-1.589	SMARCA2	-1.305

Genes	Exp Fold Change
SMARCA5	-1.306
SMARCC1	-1.365
SMN1/SMN2	-1.69
SOAT1	-1.744
SOCS1	-1.321
SOCS2	-1.707
SORT1	-1.485
SOX4	-1.513
SPAG5	-2.806
SPC25	-2.744
SPINT2	-1.568
SPOCK1	-3.713
SPP1	-1.893
SPTBN1	-1.364
SREBF1	-1.4
SREBF2	-1.591
SRPK1	-1.482
SRRT	-1.477
SRSF1	-1.576
SRSF2	-1.327
SSRP1	-1.694
STAT1	-1.525
STAT3	-1.325
STAT5B	-1.357
STIL	-2.521
STIP1	-1.348
STK17B	-1.431
STK26	-1.505
STK3	-1.32
STMN1	-1.419
SUN1	-1.357
SWAP70	-1.447
SYCP2	-1.573
TAF9B	-1.364
TAGLN2	-1.881
TCEB1	-1.606
TCEB3	-1.385
TCERG1	-1.336
TCF4	-1.309
TCF7L2	-1.458
TCP1	-1.358

Genes	Exp Fold Change
TFAM	-1.325
TFDP1	-1.523
TFDP2	-1.426
TFRC	-1.513
TGFB1	-2.053
TGFB2	-1.359
TGFBR1	-1.352
TGFBR3	-2.025
THG1L	-1.343
TJP2	-2.115
TK1	-2.042
TMOD3	-1.579
TMX1	-1.498
TNFRSF10B	-1.398
TNFRSF4	-3.199
TNFRSF8	-2.498
TNFRSF9	-1.794
TNFSF9	-1.338
TOP1	-1.379
TOP2A	-5.487
TOPBP1	-1.597
TOPORS	-1.364
TP53	-1.611
TP53I3	-1.429
TPD52	-1.326
TPX2	-2.24
TREX2	-1.513
TRIB1	-2.446
TRPC1	-1.619
TSC22D3	-1.338
TTK	-3.782
TXK	-1.546
TXN2	-1.436
TYMS	-3.511
TYROBP	-2.173
UBD	-1.888
UBE2M	-2.115
UBE2V1	-1.323
UBE2V2	-1.467
UBE4B	-1.312
UCP2	-1.595

Genes	Exp Fold Change
ULK2	-1.463
UNG	-1.324
UPF1	-1.307
USP10	-1.434
USP12	-1.322
VASP	-1.707
VAV3	-1.319
VCAN	-1.541
VCL	-2.039
VDAC1	-1.375
VEGFA	-1.575
VIM	-1.377
WAPL	-1.412
WDR48	-1.38
WEE1	-1.882
WHSC1	-1.386
WWOX	-1.531
XCL1	-1.765
YBX1	-1.476
YBX3	-2.697
YME1L1	-1.381
YWHAE	-1.988
YWHAQ	-1.346
YWHAZ	-1.369
YY1	-1.334
ZEB1	-1.381
ZFP36L2	-1.948

Up-regulated Genes in apoptosis dataset

Genes	Exp Fold Change
ADAM8	1.477
ADCK3	1.303
ADORA2A	1.313
AGA	1.346
AHI1	1.325
AKTIP	2.066
ALK	1.366
ALMS1	1.333
AMFR	1.546
ANGPTL4	1.39
APBB3	1.366
APC	1.423
AQP3	2.545
ARHGEF3	1.423
ARIH2	1.348
ATF5	1.431
ATG5	1.508
AZU1	1.311
B4GALT1	1.306
BCL2A1	1.327
BCL6	1.337
BMP1	1.365
BMP7	1.362
BRF1	1.528
BRMS1	1.321
BTG2	2.05
C3AR1	1.985
CACFD1	1.34
CACNA1A	1.301
CACNA1C	1.343
CAPN6	1.327
CAPRIN2	1.415
CASP4	1.501
CBLB	1.379
CCKBR	1.357
CCL2	1.316
CCL5	1.329
CCNL2	1.499
CCR2	2.099
CCR5	1.8

Genes	Exp Fold Change
CD2	1.535
CD44	1.426
CD47	1.32
CD59	1.414
CD69	1.775
CDCP1	1.376
CDK11A	1.319
CDKN1C	1.328
CEACAM1	1.335
CEBPB	1.67
CERS6	1.331
CLN8	1.336
CLU	1.518
CREBL2	1.305
CSF2RB	1.526
CSK	1.386
CSTB	1.499
CTSB	1.66
CX3CL1	1.367
CYLD	1.323
CYP2E1	1.366
CYP3A4	1.344
DAD1	1.31
DAO	1.333
DCT	1.336
DDAH2	1.352
DDX17	1.334
DFFB	1.498
DGCR8	1.408
DHX58	1.356
DICER1	1.343
DIDO1	1.358
DNAJC3	1.399
DNM1	1.346
DPP4	1.308
DRD2	1.346
DUSP6	1.312
DYNLL1	1.429
E2F3	1.343
E2F6	1.392
EDA	1.311

Genes	Exp Fold Change
EEF1D	1.4
EGLN3	1.688
EGR1	2.498
EHD1	1.323
EPO	1.542
EPOR	1.461
FANCF	1.336
FBLN1	1.398
FBN1	1.592
FCGR1A	1.393
FCMR	1.92
FGFR2	1.392
FIS1	1.373
FNIP1	1.504
FOXO1	1.316
FOXO3	1.306
FUBP1	1.37
FXR1	1.422
GADD45G	1.367
GAS6	1.309
GDF2	1.314
GDF5	1.341
GIMAP1-GIMAP5	1.439
GIMAP4	2.195
GIMAP5	1.439
GJB1	1.307
GLIPR1	1.353
GLO1	1.394
GLRX	1.343
GNAS	1.366
GPR65	1.386
GRIN1	1.344
GRIN2B	1.401
GRM4	1.335
GSN	1.377
HAND2	1.351
HAP1	1.323
HAS1	1.311
HBEGF	1.473
HDAC4	1.434
HFE	1.32

Genes	Exp Fold Change
HIP1R	1.368
HPCA	1.369
HSPA1A/HSPA1B	1.591
HTATIP2	1.659
HUNK	1.303
ID2	1.652
IGHG1	1.349
IGHM	1.349
IKBKB	1.435
IKZF1	1.376
IL10RA	1.512
IL17RB	1.444
IL1RN	1.406
INHA	1.404
INHBA	1.302
INPP5E	1.391
IRF7	1.978
ISG15	1.406
ITGA1	1.643
ITIH4	1.414
ITM2B	1.3
ITPR2	1.326
ITPR3	1.354
JAG2	1.535
JAK1	1.332
JUN	1.495
KAT2B	1.53
KIF1C	1.474
KIFC3	1.427
KSR1	1.332
LAMA1	1.368
LAMA3	1.512
LAMA4	1.395
LGALS3BP	1.426
LIMS1	1.509
LIMS2	1.403
LMO2	1.328
LRP5	1.552
LTBR	1.469
LYL1	1.339
MAP4	1.317

Genes	Exp Fold Change
MAP4K1	1.307
MAP4K4	1.322
MAPK11	1.315
MAPT	1.352
MBOAT7	1.344
MCOLN3	1.618
MEF2C	1.44
MGEA5	1.31
MGMT	1.34
MICAL1	1.691
mir-21	1.486
mir-22	1.952
MMP2	1.464
MPZ	1.353
MSH3	1.439
MSR1	1.312
MTOR	1.321
MZF1	1.309
NCAM1	1.335
NCK1	1.375
NCOA3	1.324
NEK1	1.337
NFIL3	1.339
NGFRAP1	1.342
NOL3	1.433
NQO2	1.769
NR1D1	1.351
NR1H3	1.749
NR2F1	1.442
NRP1	2.364
NUMA1	1.343
NUPR1	1.312
OGT	1.451
OPTN	1.312
OSGIN1	1.378
PAFAH1B1	1.375
PAM16	1.386
PCBP4	1.595
PDCD2	1.399
PDCD4	1.327
PDE4A	1.358

Genes	Exp Fold Change
PDGFB	1.344
PDHA1	1.374
PDIA2	1.381
PDLIM7	1.37
PDPK1	1.355
PGR	1.328
PHF1	1.388
PHOX2B	1.474
PIAS3	1.492
PIK3CD	1.302
PIK3IP1	1.421
PINK1	1.634
PITPNA	1.49
PKD2L2	1.503
PKM	1.858
PMEPA1	1.309
POU3F1	1.34
PPIF	1.323
PPM1A	1.303
PPP1R13L	1.555
PPP2R5C	1.468
PQBP1	1.33
PRDM16	1.529
PRDX2	1.387
PRKCG	1.319
PRKCZ	1.338
PRMT2	1.382
PTGER2	1.377
PTGS2	2.2
PTPN22	1.311
RAB28	1.39
RAD9A	1.307
RARB	1.455
RASGRP1	1.614
RBCK1	1.38
RBL2	1.494
RBM3	1.45
RBM5	1.302
RBX1	1.398
RGS4	1.341
RNF5	1.403

Genes	Exp Fold Change
RPS6KA2	1.369
RPS6KA5	1.472
S100B	1.357
SAA1	1.356
SCRIB	1.4
SDC2	1.489
SEMA4D	1.537
SFN	1.348
SFRP4	1.345
SFTPC	1.42
SH3BP2	1.335
SHBG	1.329
SIM2	1.397
SKIL	1.471
SLAMF1	1.375
SLC18A3	1.329
SLC1A3	1.332
SLC4A3	1.379
SLC4A7	1.324
SLC9A1	1.328
SMAD3	1.586
SMAD6	1.338
SMOX	1.331
SNCA	1.307
SOX10	1.307
SPDEF	1.415
SPN	1.366
SQSTM1	1.306
SRC	1.355
SRF	1.348
SRPK2	1.327
SSTR1	1.355
SSTR2	1.328
ST14	1.304
ST6GAL1	1.526
ST8SIA1	5.653
SURF1	1.315
SYNE1	1.409
TAB2	1.487
TAOK1	1.508
TARDBP	1.393

Genes	Exp Fold Change
TBX5	1.328
TBXA2R	1.339
TCF3	1.571
TDRD7	1.359
TFEB	1.365
TFR2	1.5
TGFB3	1.468
TGM1	1.404
THRA	1.351
TIA1	1.322
TIAL1	1.353
TIAM1	2.351
TIMP1	1.569
TNFRSF1A	1.371
TNFSF10	1.688
TNFSF12	1.328
TNFSF13	1.328
TNFSF8	1.526
TPM1	1.43
TRADD	1.31
TRAF5	1.378
TTN	1.323
UBA3	1.372
UBASH3A	1.384
UBE2I	1.472
UBR4	1.321
USE1	1.348
USP18	1.431
VASH1	1.33
VOPP1	1.459
VPS13A	1.337
WSB1	1.315
WWP2	1.307
XAF1	1.33
XBP1	1.468
XPC	1.333
ZBTB16	1.356
ZHX2	1.305
ZNF10	1.44
