# Supplementary figures and images for: miR-29b and miR-198 overexpression in CD8+ T cells of renal cell carcinoma patients down-modulates JAK3 and MCL-1 leading to immune dysfunction
Source: J Transl Med. 2016 Apr 11;14:84. doi: 10.1186/s12967-016-0841-9 (PMC4827202; doi:10.1186/s12967-016-0841-9)

### MCL1 RQ

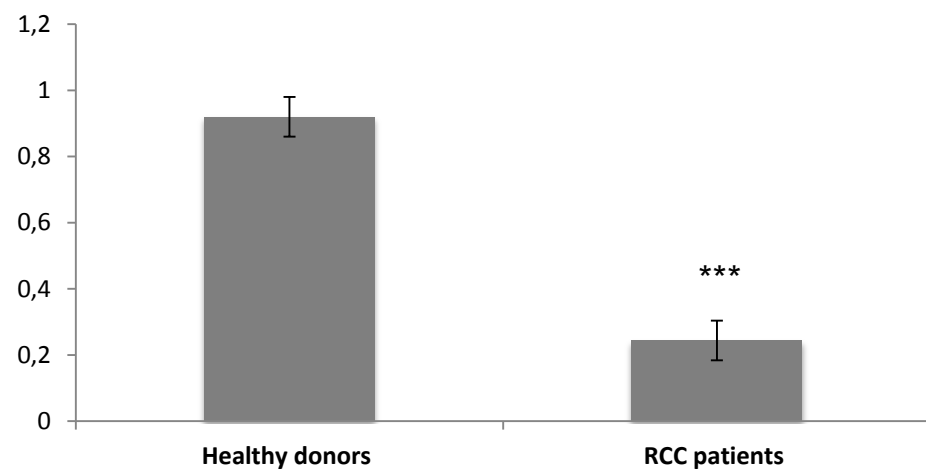

### JAK3 RQ

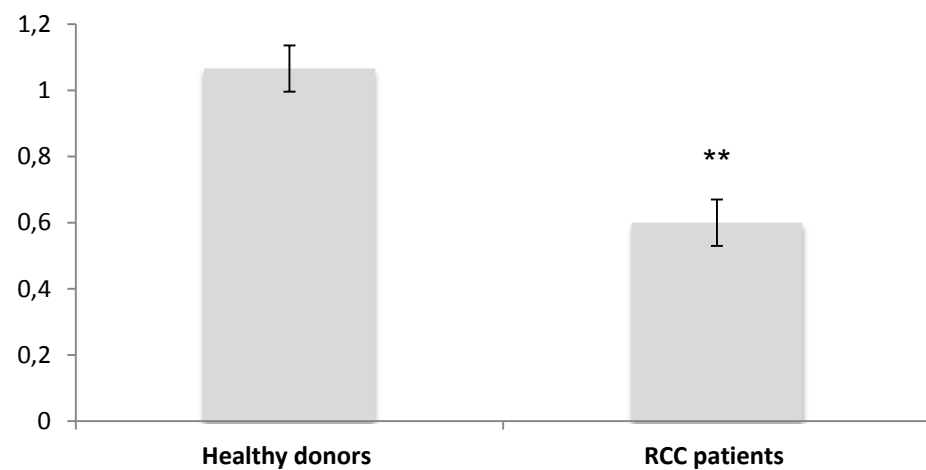

Figure S2

Supplement: Supplementary file 2 — 10.1186/s12967-016-0841-9CD8+ T cells from RCC patients exhibit downregulated MCL-1 and JAK3 gene expression. Real-time PCR analysis of MCL1 and JAK3 expression in freshly-isolated CD8+ T cells from RCC patients and healthy normal donors. *** p < 0.0001 and ** p < 0.005 for RCC patients versus healthy normal donors. [file 12967_2016_841_MOESM2_ESM.pdf]
